# The Hospital for the Insane

**Published:** 1890-02-22

**Authors:** 


### The Hospital for the Insane.

By an Official Correspondent.

The Report of the Committee on a Hospital for the Insane has been presented to the London County Council. The establishment of a hospital of 100 beds is recommended. This is to be under the divided authority of a resident superintendent and a body of visiting physicians who are to be paid officials. The former, the only specialist in Insanity, is to be responsible for the "moral"; the latter for the purely "medical" treatment of the inmates. Between these two authorities there is to be "rather a co-operation than a strict subordination." The working of this scheme will be an interesting experiment: friction is almost inevitable. Full provfsion will be made for the study of pathology.

Very distinguished gentlemen, leaders and teachers in the world of medicine and surgery, gave evidence before the committee, and from their point of view made out a strong case for the founding of such an institution. The idea is an attractive one, and may be productive of much good. The teaching of insanity is sadly neglected in our medical schools, and men are turned out quite unfitted for the responsible duties they may be called upon to undertake. The earliest stages of insanity are the least known, and are at the same time the most amenable to treatment. These come under the observation of private practitioners and of the physicians to the out-patient departments of the large hospitals; but are scarcely ever brought under the notice of the asylum officer. It is on this stage of the disease that most light is required, and this hospital, with its facilities for teaching and scientific investigation, may supply the want.

But, while willing and thankful to recognize that good results may accrue from such a hospital, one cannot but feel that the subject was hardly presented to the committee in the spirit which so great an undertaking demanded. Able men, who have spent their lives in the study of lunacy, pointed out the disadvantages of the proposed scheme, and yet no sufficient reason was seen for inviting any of them to give evidence. Not one English specialist now in practice, nor any representative of present English superintendents, was examined. This was scarcely following out the methods of scientific research to be pursued in the new institution. All the witnesses called were in harmony, and it was only apparently by accident that the views of the specialists were laid before the committee. As Mr. Carter suggests, doubtless the superintendents will accept Dr. Clifford Albutt as their exponent. Yet this gentleman's evidence is said to have been in some sense "a surprise." Are we to infer that had his opinions been known he would have been classed amongst the asylum superintendents and the few other persons of admitted authority who had a couple of skilfully-framed questions submitted to them, but were not thought of sufficient importance to be called as witnesses? These gentlemen, with Dr. Clifford Albutt, believe that drugs are not the only, and that it is doubtful whether they are the most efficacious, agents in the treatment of insanity. Yet that by all means medicines have their place. The holders of this doctrine are told that they are ignorant of the principles of modern treatment of disease. In such company they will not feel unhappy even when so stigmatized.

The skilful manner in which the witnesses were examined extorts our admiration, but the writer of the report, in his anxiety to deal a blow at those who disagree with him, makes an amusing mistake. He says 35 per cent. in asylums recovered, while only 4 per cent. are under medical treatment; therefore 31 per cent. get well, he insinuates, unnoticed and uncared for. Without discussing the correctness of his figures, which in public asylums are 10 per cent. and 40 per cent., it may be sufficient to point out that the percentage of patients under medical treatment is calculated upon the whole then-existent population, whereas the recoveries are calculated upon the yearly admissions. Thus, at one time a number of patients equal to the total of recoveries in the year may be under medical treatment.

He also draws a distinction between "recovery" and "cure"; again conveying a covert sneer at the present methods of treatment. A patient "recovers" if everything possible with regard to diet and environment is done for him; but is "cured" if drugs are administered. The physician who steers a patient safely through typhoid without the use of drugs is less skilful than one who "gives medicine out of a bottle."' He says also that conditions which have unquestionably been shown by experience to be of great advantage in promoting "recovery," would not of necessity be equally important with regard to "cure." Arguments must be very scarce when a case has to be bolstered up with such statements as the above.

We, however, welcome the Hospital for the Insane, and wish its promoters God speed. Let us hope their sanguine expectations will be fulfilled. It is unfortunate that they have begun the good work in such a partisan spirit. The committee, by not calling gentlemen conversant with the practical difficulties of the question, have lost much useful information which might have enabled them to avoid many difficulties, and would have assisted them in overcoming others.

[We publish without comment this criticism of the report of the Committee of the London County Council by a correspondent who represents the "official" view. We propose to give the substance of this very able report in an early issue, and shall be glad to open our columns to a full discussion of the questions involved.—Ed. *T. H.*]

